# Polysaccharide-Based Carriers for Pulmonary Insulin Delivery: The Potential of Coffee as an Unconventional Source

**DOI:** 10.3390/pharmaceutics15041213

**Published:** 2023-04-11

**Authors:** Sara A. Valente, Guido R. Lopes, Isabel Ferreira, Miguel F. Galrinho, Margarida Almeida, Paula Ferreira, Maria T. Cruz, Manuel A. Coimbra, Cláudia P. Passos

**Affiliations:** 1 LAQV-REQUIMTE, Department of Chemistry, University of Aveiro, 3810-193 Aveiro, Portugal; 2 Center for Neuroscience and Cell Biology, University of Coimbra, 3004-517 Coimbra, Portugal; 3 Faculty of Pharmacy, University of Coimbra, 3000-548 Coimbra, Portugal; 4 CICECO, Department of Materials and Ceramic Engineering, University of Aveiro, 3810-193 Aveiro, Portugal

**Keywords:** drug delivery, protein delivery, microparticles, arabinogalactans, galactomannans, inhalation

## Abstract

Non-invasive routes for insulin delivery are emerging as alternatives to currently painful subcutaneous injections. For pulmonary delivery, formulations may be in powdered particle form, using carriers such as polysaccharides to stabilise the active principle. Roasted coffee beans and spent coffee grounds (SCG) are rich in polysaccharides, namely galactomannans and arabinogalactans. In this work, the polysaccharides were obtained from roasted coffee and SCG for the preparation of insulin-loaded microparticles. The galactomannan and arabinogalactan-rich fractions of coffee beverages were purified by ultrafiltration and separated by graded ethanol precipitations at 50% and 75%, respectively. For SCG, galactomannan-rich and arabinogalactan-rich fractions were recovered by microwave-assisted extraction at 150 °C and at 180 °C, followed by ultrafiltration. Each extract was spray-dried with insulin 10% (*w*/*w*). All microparticles had a raisin-like morphology and average diameters of 1–5 µm, which are appropriate for pulmonary delivery. Galactomannan-based microparticles, independently of their source, released insulin in a gradual manner, while arabinogalactan-based ones presented a burst release. The microparticles were seen to be non-cytotoxic for cells representative of the lung, specifically lung epithelial cells (A549) and macrophages (Raw 264.7) up to 1 mg/mL. This work shows how coffee can be a sustainable source of polysaccharide carriers for insulin delivery via the pulmonary route.

## 1. Introduction

Diabetes mellitus is a chronic disease that affects millions of people worldwide. The most common forms are type 1 diabetes, associated with complete insulin deficiency, and type 2 diabetes, characterised by the gradual loss of insulin secretion (frequently on the background of insulin resistance) [1]. Currently, the most used methodology for insulin delivery is subcutaneous injection, which is invasive and can cause discomfort, pain, bruises, and even local infection. Usually, several administrations a day are required, which is very inconvenient and may lead to patient noncompliance. Several non-invasive alternatives are being developed [2,3], and pulmonary delivery has been shown to be one of the most promising routes. 

Pulmonary delivery is the inhalation of a drug formulation through the respiratory system with later deposition and absorption at the lower airways [4]. Because lungs are highly vascularised and display a particularly large surface area (>100 m^2^) and very thin epithelium at the alveoli (<1 µm), the pulmonary route is one of the most rapidly absorbing pathways. In addition, the low concentration of enzymatic activity at this site also allows the safe passage of insulin to circulation without degradation [5]. One of the biggest challenges in pulmonary delivery resides in the success of drug formulations in reaching the lower airways. It has been shown that to reach the deep lung, drug formulations must be in the form of micron-sized particles, particularly with aerodynamic diameters between 1 and 5 µm [6]. 

Powdered particle preparation methodologies often require conditions such as high temperature, pH changes, shear stress, or even the presence of metals that may compromise the integrity of proteins such as insulin. To avoid degradation, polymers are often used to protect the bioactive form of the protein during powder preparation and storage [7]. In addition, the incorporation of insulin in polymeric microparticles can also provide a way to manipulate the rate of insulin delivery, resulting in a formulation with controlled release. The rate at which insulin is liberated from particles is especially important because a fast and large (i.e., burst) release can lead to hypoglycaemic episodes, which are associated with several complications and even death [8], whereas a delayed release may lead to hyperglycaemia, which is also associated with negative effects [9]. The proof of concept for inhalable insulin has been made with some formulations that have reached the market: Exubera™ (discontinued in 2007) and Afrezza™ (currently available since 2014). Although both formulations present several possible side effects for patients presenting lung-related diseases [10,11,12], pulmonary delivery is a convenient solution for most diabetic patients. The appearance of alternative formulations, such as those based on polysaccharides, may present an opportunity for new inhalable insulin formulations. 

Polysaccharides are naturally abundant, biocompatible, and biodegradable polymers. They are extensively used as carriers in drug delivery [13], including pulmonary [4]. The structural diversity within the polysaccharide family allows a carrier to be chosen for the desired function. Galactomannans are polysaccharides that have been used as carriers for pulmonary delivery [14] and are obtained from different sources, such as the locust bean or guar gums. Galactomannans are composed of a backbone of (β1→4)-linked d-mannose with single (α1→6)-linked d-galactose side chains. According to the literature, coffee is another source of galactomannans. Roasted coffee beans’ largest constituents are polysaccharides, including galactomannans and arabinogalactans [15]. Arabinogalactans are composed of a backbone of (β1→3)-linked d-galactose with (β1→6)-linked side chains, which, in turn, can be substituted at the O-3 position with (α1→5)-linked l-arabinose side chains. These polysaccharides are both “Generally Recognised As Safe” by the FDA [16,17] and EFSA [18,19] and have been used in the food, cosmetics, and pharmaceutical industries [20,21]. Coffee, which is one of the most transacted commodities, suffers extensive processing from the plant to the bean and to coffee beverage preparation, which results in high amounts of wasted by-product materials [22,23]. Toward the development of a sustainable green bioeconomy, these by-products are an opportunity for polysaccharide extraction, as most of them are still rich in several biopolymers, which otherwise would be discarded or used for menial applications such as fertilisers [24]. Roasting of the seeds decreases the polymerisation and branching of the polysaccharides, increasing their solubility and facilitating polysaccharide extraction [25]. Most of these polysaccharides remain in the spent coffee grounds (SCG), from which they can be recovered upon the use of extraction technologies, such as microwave irradiation [26,27,28,29]. 

The hypothesis that led to the development of the present work was that galactomannans and arabinogalactans extracted from coffee could be used as carriers for pulmonary insulin delivery. These polysaccharides have high-water solubility and temperature stability, independently of pH. For that, galactomannan-rich and arabinogalactan-rich fractions were purified from coffee beverages. For a more sustainable approach, these polysaccharides were also recovered from SCG using microwave-assisted extraction before being further purified. All these extracts were used to prepare insulin-loaded microparticles by spray drying. These particles were characterised for their physicochemical and biological properties. 

## 2. Materials and Methods

### 2.1. Sample Preparation

Polysaccharides were obtained from instant coffee powder by dissolving 40 g in 2 L of hot (80 °C) distilled water under magnetic stirring for 10 min. Espresso coffee was also used as a source of polysaccharides. Fifty espresso coffees were prepared using an espresso machine. For each coffee, approximately 6 g of roasted coffee powder and 40 mL (±2 mL) of distilled water were used. Both samples were stored at 4 °C until further use.

The samples were vacuum filtered with 1.2 µm glass microfiber filters to remove any solid materials. Ultrafiltration was conducted with a Millipore Labscale TFF System (Millipore, Darmstadt, Germany) with a membrane of 5 kDa cut-off pores (Pillicon^®^ XL—Millipore), allowing the high molecular weight material (HMWM) to be obtained in the retentate. To guarantee that all low molecular weight materials were removed (eliminating any possible adsorption effects [30]), dialysis was performed with a 12 kDa cut-off membrane (Medicell Membranes Ltd., London, UK). Dialysis water was changed twice a day until a conductivity below 20 µS/cm^2^ was obtained. Every time, a few drops of toluene and chloroform were added to prevent microorganism growth. After dialysis, the retentate was centrifuged at 15,000 rpm for 15 min at 4 °C to obtain the Water-Soluble Supernatant (WSSn).

The WSSn from both samples were further subjected to 2 sequential ethanol precipitations. These were performed with 50% and 75% of ethanol for the separation and purification of galactomannans and arabinogalactans, respectively [31,32]. A mixture of WSSn:ethanol in a 1:1 ratio was magnetically stirred for 5 min at room temperature and allowed to precipitate for 1.5 h at 4 °C. After, the mixture was centrifuged at 5000× *g* for 30 min at 4 °C, the 50% ethanol precipitate (PpEt50) was obtained. The precipitate was rotary evaporated, frozen, and freeze-dried. The 50% ethanol supernatant (SnEt50) was then submitted to 75% ethanol precipitation upon the addition of another volume of ethanol (final proportion of 1:3 *V*/*V* WSSn:ethanol). Again, the mixture was magnetically stirred for 5 min at room temperature and allowed to precipitate for 1.5 h at 4 °C. Afterward, the mixture was centrifuged at 5000× *g* for 30 min at 4 °C. Both the 75% ethanol precipitate (PpEt75) and the 75% ethanol supernatant (SnEt) were rotary evaporated, frozen, and freeze-dried.

The spent coffee grounds (SCG) that resulted from espresso coffee preparation were also used as a polysaccharide source. The SCG were oven dried at 105 °C overnight and stored in a desiccator at room temperature until further use. Polysaccharides were extracted using microwave irradiation performed with a MicroSYNTH Labstation (maximum output 1 kW, 2.45 GHz, Milestone Srl., Bergamo, Italy) and high-pressure 100 mL polytetrafluoroethylene reactors. Suspensions containing 1:10 (g:mL) of SCG and distilled water were prepared in a total volume of 60 mL for each reactor. Two experiments with 4 reactors each were performed at 150 °C and another 2 reactors at 180 °C. Each experiment started at room temperature with a heating ramp of 25 °C/min, followed by 2 min of isocratic heating at temperatures of 150 or 180 °C [28]. After, the reactors were allowed to cool down to room temperature. The suspensions were vacuum filtered with 1.2 µm glass microfiber filters. The insoluble material was discarded. The soluble material was subjected to ultrafiltration (5 kDa) to recover the retentates, MW150 retentate, and MW180 retentate, respectively, for the 150 °C and 180 °C conditions, as previously described.

### 2.2. Sugar Analysis

All fractions were assessed for their polysaccharide content and the corresponding monomeric residue composition by gas chromatography in the form of alditol acetates. For that, 1–2 mg of the sample was hydrolysed with H_2_SO_4_ 2 M for 1 h at 120 °C. 2-Deoxyglucose was added as an internal standard (2 mg/mL). Derivatisation and analysis were performed as described by Passos et al. [27].

### 2.3. Microparticle Preparation & Characterisation

#### 2.3.1. Spray Drying

The purified fractions of PpEt75 from instant coffee, PpEt50 from espresso coffee, and MW150 retentate and MW180 retentate from SCG were spray dried in the presence of insulin (10%, *w*/*w*) in order to obtain microparticles. For that, 270 mg of the freeze-dried sample was dissolved in distilled water at 80 °C under magnetic stirring. The solutions were allowed to cool down to room temperature. Once the sample was below 40 °C, 30 mg of insulin (human recombinant, Cat# 91077C, Sigma Aldrich, St. Louis, MO, USA) previously dissolved in 1 mL of HCl 0.01 M was added, obtaining a mixture with 10% (*w*/*w*) insulin. The pH of the solution was previously adjusted to 3–4 (below the isoelectric point of insulin—pH 5.4) with HCl 1 M to guarantee the full dissolution of insulin. The volume was then adjusted to 100 mL with distilled water (final concentration of mixture 3 mg/mL, with insulin at 0.3 mg/mL). The mixtures were spray dried using a Büchi Mini Spray Dryer B-190 (Büchi Labortechnik AG, Flawil, Switzerland) equipped with a high-efficiency cyclone. The operating parameters were as follows: inlet temperature of 150 °C, aspirator to 95%, pump to 12%, and flow rate at 3.6 mL/min. These conditions resulted in an outlet temperature of 80 °C. After spray-drying, microparticles were collected using a paintbrush and were stored in a desiccator until further use. The spray-drying yield was calculated by dividing the mass of the spray-dried material by the total mass of solids in the mixture. The loading capacity and association efficiency were determined as described by Grenha et al. [33]. 

#### 2.3.2. Scanning Electron Microscopy (SEM)

The spray-dried powders were placed in a stainless-steel support with a conductive copper strip. Samples were coated using a carbon rod coater Emitech K950X, which deposited a thin carbon layer on the surface of the microparticles. The particles were observed in a scanning electron microscope Hitachi, SU-70 (Tokyo, Japan) operated at 15 kV. Images were treated using the ImageJ software (ImageJ 1.53k) [34]. The particle size distribution was determined after quantifying the volume of at least 50 particles in the micrographs. The diameter of each particle was collected using the broadest dimension (the particles were not perfectly spheric), and a volume of a sphere was considered. Intervals of 0.5 µm diameter length were used for the estimation of the distribution of particle volume. The total volume occupied by the particles was calculated by the sum of the volume of all particles. 

### 2.4. In Vitro Insulin Release

To assess the insulin release rate from the microparticles, release assays consisting of at least two independent experiments per sample were performed based on the methodology of Alves et al. [14] with alterations. A solution of 0.01 M phosphate-buffered saline (PBS, pH 7.4) and 1% (*V*/*V*) of Tween80^®^ (PBS-T80) was used to simulate the lung fluid. After weighing 9 mg of each microparticle sample into 20 mL containers, 9 mL of the previously prepared PBS-T80 was gently transferred to each container. The samples were incubated in an orbital shaker at 37 °C with 100 rpm. A 1 mL aliquot was immediately collected, representing the initial time. Additional aliquots were taken at 5, 10, 20, 30, 40, 50, and 60 min. Each aliquot was then centrifuged (Thermo Scientific Multifuge X1R, Waltham, MA, USA) at 10,000× *g* for 5 min, followed by syringe filtration using GDX (Whatman) filters with a pore size of 0.2 µm and 13 mm of diameter. To assess the total entrapped insulin, 1–2 mg of microparticles were weighed, and the volume of PBS-T80 was adjusted to 1 mg/mL. Then, the microparticles were incubated under vigorous magnetic agitation at 37 °C for 60 min. The sample was centrifuged at 10,000× *g* for 5 min, followed by 0.2 µm filtration.

Insulin in the filtered supernatants was quantified in a High-Performance Liquid Chromatographer (HPLC) with a Diode Array detector model Ultimate 3000 (column C18 column LiChrospher^®^ RP-18, 5 μm particle size, I.D. 25 cm × 4.6 mm, end-capped), based on the chromatographic separation methodology of Amidi et al. [35], with some modifications. The column was initially equilibrated for 15 min with a solvent mixture consisting of 70% Eluent A (0.1% trifluoroacetic acid in H_2_O) and 30% Eluent B (100% acetonitrile) at a flow rate of 1 mL/min and an injection volume of 20 µL. The run was set to start with the conditions of the equilibration (70% Eluent A and 30% Eluent B) following a gradient decrease in eluent A to 60% for 9 min. These conditions were held for 2 min, after which they returned to the initial conditions in a gradient for 2 min and were held for another 2 min, finishing the run (total of 15 min). The column temperature was set to 37 ± 2 °C. The insulin peak was quantified at 200 nm using a calibration curve obtained from standard solutions with concentrations in the range of 0 to 200 µg/mL (R^2^ = 0.9928, Figure S1 of supplementary material).

The filtered supernatant was also used for an absorbance reading at 405 nm, characteristic of a brown colour. A quartz 96-well microplate (Hellma Analytics, Müllheim, Germany) was used. All samples were diluted at a ratio of 1:4 in PBS-T80. Then, 150 µL of each diluted sample was transferred to each well of the microplate. Absorbances were read using a Biotek EON UV/Vis spectrophotometer (BioTek Instruments, Inc., Winooski, VT, USA).

### 2.5. Cytotoxicity Assay

Cytotoxicity assays were performed in two cellular lines: the murine-leukemic monocyte-macrophage cell line RAW 264.7 (ATCC Cat# TIB-71, RRID:CVCL_0493) and the human lung adenocarcinoma alveolar epithelial cell line A549 (ATCC Cat# CCL-185, RRID:CVCL_0023). The number of microparticles to be tested was initially selected based on the results reported in the literature, in which microparticles were studied in concentrations ranging from 0.1 to 1 mg/mL [36,37]. We then adjusted the concentration of the particles to be tested according to the cytotoxicity results in A549. The results herein presented are from microparticles loaded with 10% of insulin. Because insulin has been reported not to be cytotoxic [38], only insulin-loaded particles were tested concerning cytotoxicity.

The RAW 264.7 cell line was cultured in a DMEM medium pH 7.4, supplemented with 10% foetal bovine serum (FBS), 25 mM glucose, 18 mM sodium bicarbonate, 100 U/mL penicillin, and 100 μg/mL streptomycin. The A549 cell line was cultured in a DMEM medium pH 7.4, supplemented with 10% heat-inactivated FBS, 25 mM glucose, 44 mM sodium bicarbonate, 100 U/mL penicillin, and 100 μg/mL streptomycin. Cells were kept at 37 °C in a 5% CO_2_ humidified atmosphere. Sub-culturing was performed according to the manufacturer’s recommendations. Morphological cell alterations were monitored by microscope observation. 

Cell viability was assessed using the resazurin reduction assay, as previously described [39], with minor modifications. Raw 264.7 and A549 cells were seeded in 96-well plates (200 μL/well) at a concentration of 50,000 and 15,000 cells/well, respectively, and incubated overnight to allow their attachment to the surface of the plates. The cells were then exposed to different concentrations of the different microparticles. Microparticle solutions were prepared by dissolving the dried microparticles in DMEM media. A volume of 200 μL of each microparticle suspension was added to each well. Upon 24 h of incubation, the medium was replaced with 100 μL of fresh medium supplemented with 50 μM resazurin sodium salt, and the cells were further incubated for an additional 1.5 h (Raw 264.7) or 2 h (A549). At this point, the fluorescence intensity of resorufin, which can only be reduced from resazurin by viable cells, was detected at 544 nm (excitation) and 590 nm (emission), using the Synergy™ HT plate reader (BioTek Instruments, Inc., Winooski, VT, USA). The relative viability of the cells was calculated as follows: Relative viability (%) = (Sample fluorescence intensity/Control fluorescence intensity) × 100%.

### 2.6. Statistical Analysis

Cellular assays were carried out in three independent experiments. One-way ANOVA with Dunnetts’s multiple comparisons test was performed to compare the microparticles’ treatment with untreated cells (Ctrl). All analyses were conducted using GraphPad Prism version 8 for macOS (GraphPad Prism, RRID:SCR_002798, San Diego, CA, USA).

## 3. Results and Discussion

In this work, coffee was used as a source of polysaccharides that are suitable for the delivery of insulin by the pulmonary route. Espresso coffee was used as a source of galactomannan-rich fractions, whereas instant coffee was the source of arabinogalactans. In addition, spent coffee grounds (SCG) were used as a source of both polysaccharides, depending on the extraction conditions. Hereinafter, samples are identified according to the type of coffee from which they originate: instant (I), espresso (E), and SCG (S). Purification processes, which included ultrafiltration and, in some cases, additional dialysis steps, were performed to completely remove active compounds such as phenolics, alkaloids, and sugars [30], which allowed us to obtain inert carriers. Furthermore, ethanol precipitations were also performed to recover the polysaccharide-rich fractions and remove melanoidin (nitrogenated high molecular weight brown-coloured compounds mostly soluble in the ethanol fraction, SnEt75), which were considered to be very active as well [40].

### 3.1. Samples Preparation and Characterization 

**Figure 1:**
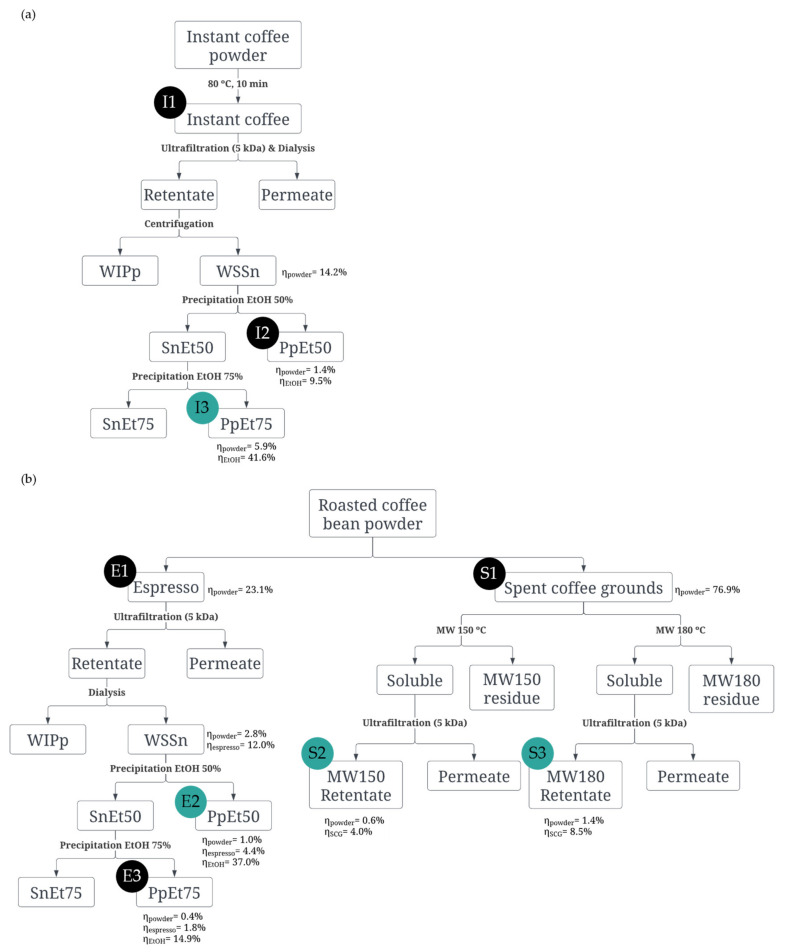
Polysaccharide purification process scheme from (**a**) Instant coffee; (**b**) Espresso coffee and spent coffee grounds.

**Figure 2:**
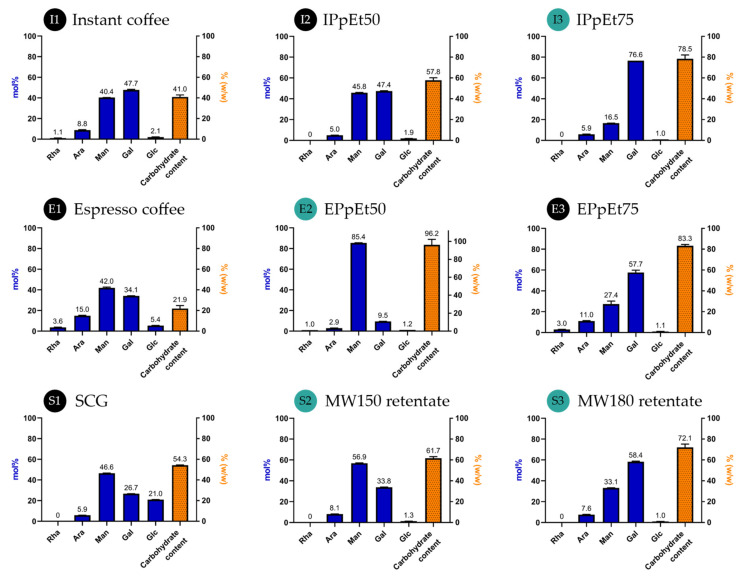
Carbohydrate content and composition of chosen fractions. E1, E2, and E3 correspond to fractions from espresso coffee; I1, I2, and I3 correspond to fractions from instant coffee, and S1, S2, and S3 correspond to fractions from SCG. Codes names are referenced in Figure 1.

Figure 1 shows the schematic diagrams of the purification of polysaccharides, whereas Figure 2 presents the monomeric carbohydrate composition of the polysaccharides and their purity in the fractions. Yields were calculated in relation to the mass of powder (η_powder_). Additionally, the yield of the ethanol precipitated fractions (η_EtOH_) was calculated in relation to the mass of soluble solids in the water-soluble supernatant (WSSn) fraction obtained after ultrafiltration and dialysis of I and E (Figure 1).

The instant coffee used was composed of 41.0% (*w*/*w*) of carbohydrates, exhibiting mainly galactose (47.7 mol%), mannose (40.4 mol%), and arabinose (8.8 mol%) (Figure 2, I1). This monomeric carbohydrate composition is diagnostic of the occurrence of galactomannans and arabinogalactans [41]. The ultrafiltration and dialysis steps, followed by ethanol fractionation, allowed a polysaccharide fraction to be obtained, which was recovered by precipitation in 50% ethanol (IPpEt50, I2), accounting for 1.4% of the initial powder and a fraction recovered by precipitation in 75% ethanol (IPpEt75, I3), accounting for 5.9% of the initial powder (Figure 1a). Both fractions resulted in an increase in polysaccharide content. The IPpEt50 (Figure 2, I2) presented a similar composition to the pristine sample, whereas the IPpEt75 (Figure 2, I3) was enriched in galactose (76.6 mol%), with smaller amounts of mannose (16.5 mol%), and arabinose (5.9 mol%), which are characteristic of the prevalence of arabinogalactans [42]. The persistent brown colour showed that this fraction also contained melanoidins, which are a high molecular weight nitrogen-containing brown compounds characteristic of roasted formulations [40]. The yield and composition of the IPpEt75 fraction showed that instant coffee was a source of arabinogalactans. 

The espresso coffee sample was composed only of 21.9% (*w*/*w*) of carbohydrates, mainly mannose (42.0 mol%), galactose (34.1 mol%), and arabinose (15.0 mol%), also containing glucose and rhamnose in smaller amounts (Figure 2, E1) in accordance with the literature [31,32]. The EPpEt50 fraction, recovered after ultrafiltration, dialysis, and fractionation with 50% ethanol, was composed mainly of carbohydrates (96.2%), showing an increase of more than 4-fold in comparison with the espresso sample. Mannose accounted for 85.4 mol%, 9.5 mol% was galactose, and 2.9 mol% was arabinose (Figure 2, E2) as a composition characteristic of pure galactomannans [31,43], justifying its use despite its low yield. On the contrary, fraction EPpEt75 (Figure 2, E3), which was rich in carbohydrates (83.3%), was composed mainly of galactose (57.7 mol%), with a profile characteristic of a mixture containing arabinogalactans and a smaller proportion of galactomannans. 

The SCG resultant of the espresso coffee preparation (Figure 1b) represented 76.9% of the powder used. The carbohydrate content of SCG was 54.3% (Figure 2, S1), about twice as much as the espresso coffee, confirming that most of the carbohydrates in the coffee powder remained in the residue [27]. It was composed mainly of mannose (46.6 mol%) and galactose (26.7 mol%) from galactomannans and arabinogalactans and of glucose (21.0 mol%) from cellulose [26]. The fraction obtained by the microwave-assisted extraction (MW) at 150 °C and ultrafiltration (MW150 retentate, Figure 1, S2) had a carbohydrate content (61.7%) that was higher than that of the SCG and was also enriched in the sugar residues characteristic of galactomannans (Figure 2, S2). The fraction obtained with MW at 180 °C (MW180 retentate, Figure 1, S3) had an even higher carbohydrate content (72.1%) than that of the MW150 retentate (Figure 2, S3), which was composed mostly of galactose (58.4 mol%), followed by mannose (33.1 mol%) and arabinose (7.6 mol%). This composition is suggestive of arabinogalactans’ prevalence with the presence of some content of galactomannans. These results are in line with the literature, where it was seen that at 150 °C galactomannans were more extractable than arabinogalactans from SCG and, at 180 °C, arabinogalactans were more extractable than galactomannans [28,44]. 

Based on the results obtained, fraction IPpEt75 was selected as an arabinogalactan-rich sample, and fraction EPpEt50 was selected as a galactomannan-rich sample for particle formation. Additionally, to meet a more sustainable use of raw materials, the two fractions recovered from SCG were also used.

### 3.2. Microparticles Formation and Characterization 

**Figure 3:**
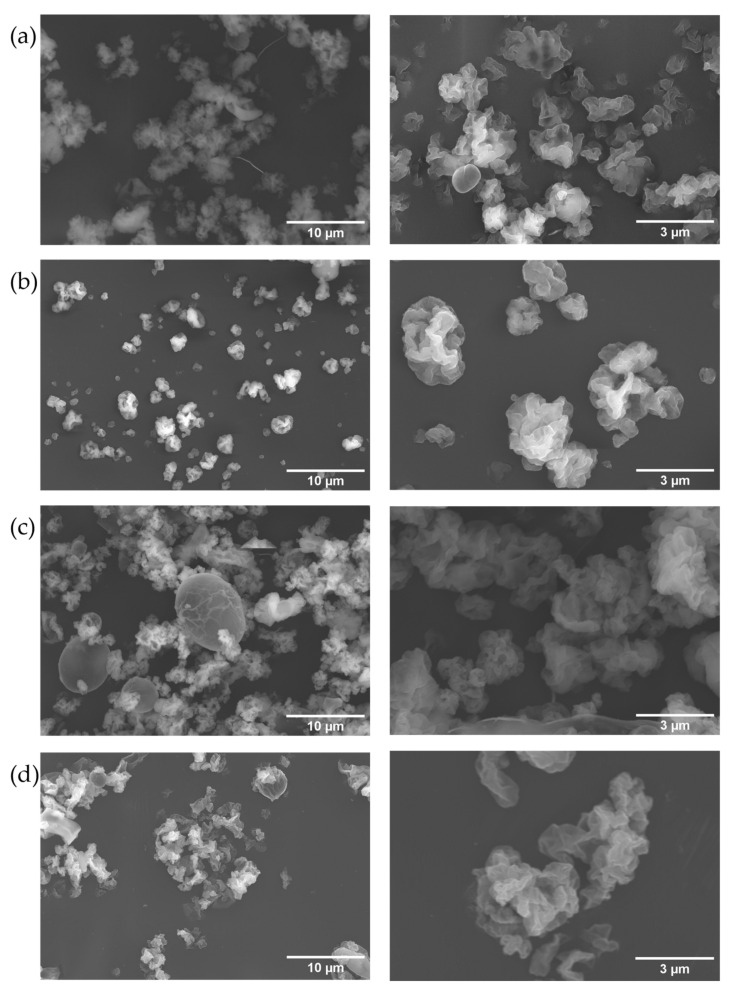
SEM micrographs of particles made with: (**a**) IPpEt75; (**b**) EPpEt50; (**c**) MW150 retentate; and (**d**) MW180 retentate. Images on the left have an ampliation of 3 k and on the right 10 k. Images on the left and on the right were collected with a 3000- and 10,000-times magnification, respectively.

Considering that, in all cases, a mass ratio of polysaccharide/insulin (*w*/*w*) of 10:1 was used, the recovered total mass yield was 63–73%, with an association efficiency for insulin resembling 75–88%, and a corresponding loading capacity of 12%. The morphology of the particles resulting from the spray drying was studied by scanning electron microscopy analysis (Figure 3). The particles presented a raisin-like shape. This type of morphology should be advantageous for applications in pulmonary delivery as it was reported that the roughness of the surface increased the drag force, thus producing better flowability on the airways [45,46]. This has also been observed in insulin particles [47].

**Figure 4:**
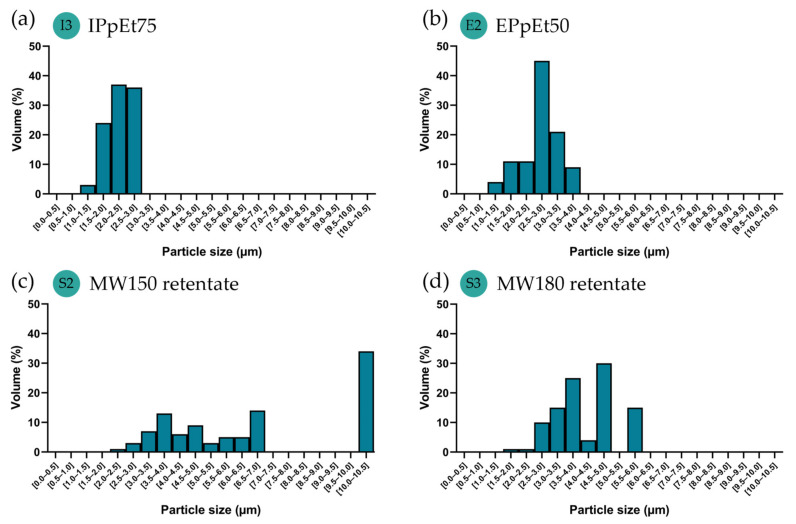
Particles volume distribution histograms based on the 2D particle size quantified in the SEM micrographs, correspondently made with (**a**) IPpEt75, (**b**) EPpEt50; (**c**) MW150 retentate, and (**d**) MW180 retentate. Images were treated using the ImageJ software (ImageJ 1.53k).

**Figure 5:**
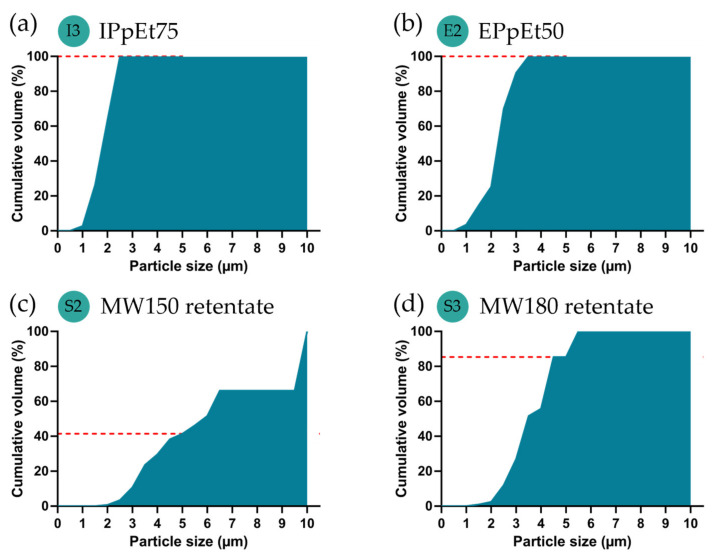
Cumulative volume determined based on the particle size for microparticles made with (**a**) IPpEt75, (**b**) EPpEt50; (**c**) MW150 retentate; and (**d**) MW180 retentate.

The particle size between 1 and 5 µm has been reported to reach the alveoli more likely and, therefore, have better efficiency for pulmonary delivery [6]. Despite the heterogeneity of particle sizes that could be found (Figure 4), most of them were distributed within the desired range and could, therefore, be considered for the expected effect and analysed in a cumulative way (Figure 5). Both IPpEt75 and EPpEt50-based microparticles had 100% of their population below de 5 µm, with only a very small percentage below 1 µm (Figure 5a,b). The MW150 retentate-based microparticles presented only 42% of its population within 1–5 µm (Figure 5c), which may be due to the contribution of the high dimensions (8–10 µm) of egg-like particles (Figure 3c). The MW180 retentate-based microparticles presented 85% of its population within the 1–5 µm range (Figure 5d). Therefore, it can be expected that the microparticles obtained will be able to reach the alveoli.

### 3.3. Insulin In Vitro Release

**Figure 6:**
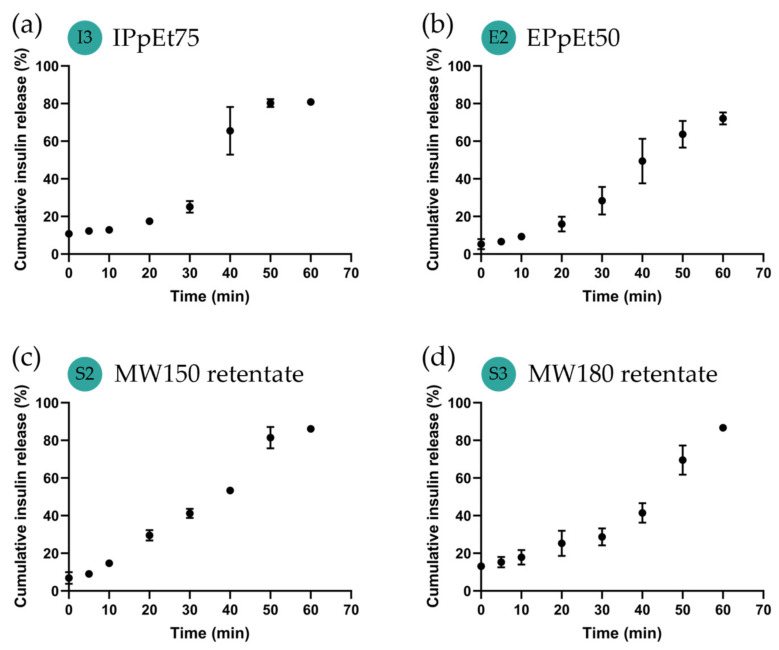
Insulin in vitro release from microparticles based on (**a**) IPpEt75, (**b**) EPpEt50; (**c**) MW150 retentate; (**d**) MW180 retentate. Insulin was quantified using HPLC. Data correspond to the mean ± SEM of at least two independent experiments.

The rate of drug release from the carriers must be carefully analysed in new formulations because different release kinetics dictate the corresponding outcome of the drug. Figure 5 shows that insulin was totally released in a time-dependent manner for all the samples. This means that to reach 0.35 mg of insulin, the average amount required by a patient per meal [48], 3–4 mg of powder of the proposed formulation would be necessary to reach the range of therapeutic dosage. The profile of release differed from sample to sample. For the IPpEt75-based microparticles, the release presented a sharp sigmoidal profile, with a burst release between 30 and 40 min, reaching 81% of insulin at 1 h (Figure 6a). From the EPpEt50-based microparticles, insulin was released gradually, reaching about 72% of the total release at 1 h (Figure 6b). Regarding the microparticles assembled with extracts of the SCG, MW150 retentate-based microparticles were released in a linear manner (R^2^ = 0.9620) throughout the time analysed for up to 86% of the insulin content (Figure 6c). From the MW180 retentate-based microparticles, the release profile resembled a smooth sigmoid, with a very slow release in the first 30 min and a gradual release in the following 30 min, which also reached about 86% of the insulin content (Figure 6d). 

Polysaccharide dissolution is a hydration process where polysaccharide–polysaccharide interactions are replaced by polysaccharide–water interactions. When the microparticles were prepared by spray-drying, the polysaccharide organisation became structured and compact, hindering the penetration of water inside the powder and behaving similarly to a hydrophobic material [49]. This can happen because arabinogalactans can promote hydrophobicity due to the associated proteins [40,43], thus hindering water penetration. Meanwhile, the solvent creates an envelope around the polymer chains [50]. However, once water diffuses that barrier, due to the high solubility of arabinogalactans, the structure rapidly disintegrates, and the insulin release rate accelerates. This effect on release was seen with IPpEt75-based microparticles. A correlation (R^2^ = 0.9731) between the release of insulin determined by HPLC and the absorbance at 405 nm, with the diagnostic of the brown colour of the medium, was observed (Figure S2a of supplementary material), as well as the complete release of insulin content. This allowed us to conclude that there was a relationship between the disintegration of the particle and the release of insulin, with no interference from the matrix. Although a faster release may approach the same effect of a burst release, which is not recommended for drug delivery [51], in the case of insulin, a fast release can be useful in prandial periods [52]. 

The release kinetics of insulin from MW150 retentate-based microparticles (Figure 6c) resembles the one from EPpEt50-based microparticles (Figure 6b), which can be explained by their similar polysaccharide composition, which is richer in galactomannans. As the water diffuses into the microparticle, the hydrophilicity of the galactomannans promotes the interaction with water and the dissolution of insulin. However, because the galactomannans were not easily dissolved, and the water penetration was slow, insulin release occurred in a gradual manner. As it was observed for the IPpEt75-based microparticles, a correlation (R^2^ = 0.8380, Figure S2b of supplementary material) between the release of insulin and the absorbance at 405 nm of the medium was observed. This allowed us to conclude that, similarly to the arabinogalactans, the galactomannan-based microparticles presented a relationship between the particle's disintegration and the insulin release. This release profile was reported as being advantageous to maintain basal insulin levels [53]. The smooth sigmoid insulin release seen from the MW180 retentate-based microparticles can be explained because the extract used to make these microparticles was a mixture of galactomannans and arabinogalactans. Thus, both polysaccharides contributed significantly to insulin release behaviour.

### 3.4. Cytotoxicity Assay

**Figure 7:**
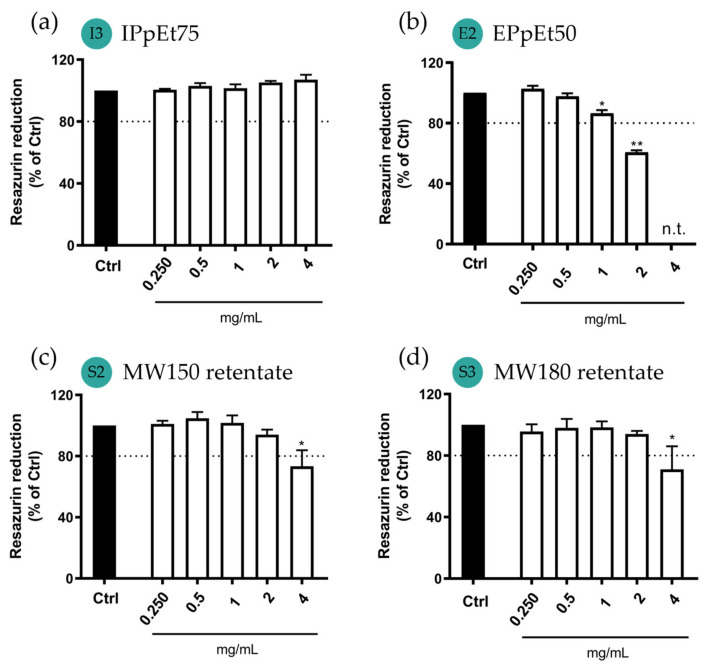
Effect of microparticle exposure for 24 h on cellular viability of human alveolar epithelial cells (A549 cell line) using microparticles made from (**a**) IPpEt75; (**b**) EPpEt50; (**c**) MW150 retentate; and (**d**) MW180 retentate. n.t.—not tested. Data correspond to the mean ± SEM of at least three independent experiments performed in duplicate and are represented as a % of untreated cells (Ctrl). Statistical analysis: one-way ANOVA with Dunnett’s multiple comparison test; Significance levels are as follows: * *p* < 0.01, and ** *p* < 0.001 compared to Ctrl.

As the microparticles are expected to reach the deep lung, it is of the utmost importance to evaluate their cytotoxicity toward cells representative of the lung, specifically human pulmonary epithelial cells (A549) and macrophages (Raw 264.7) (Figure 7). Microparticles were tested in concentrations ranging from 0.250 mg/mL to 4 mg/mL. Overall, all the microparticles tested showed no cytotoxicity at concentrations equal to or below 2 mg/mL, with the exception for microparticles made with EPpEt50. In A549 cells, the microparticles made with the IPpEt75 showed no reduction in cell viability for all the concentrations tested (Figure 7a), thus suggesting non-cytotoxicity for lung cells. The microparticles made with EPpEt50 (Figure 7b) were shown to be cytotoxic (61% cell viability; *p* < 0.0001) at the highest soluble concentration (2 mg/mL). At 4 mg/mL, the insolubility of the EPpEt50-based microparticles made it impossible to test cell viability at this concentration. At 1mg/mL, cell viability reached 87%, although their difference to untreated cells remained statistically significant (*p* < 0.01). Nevertheless, given their low statistical difference and since cell viability was above 80%, microparticles made with EPpEt50 were considered non-cytotoxic at concentrations of 1 mg/mL and below. Cells treated with microparticles made from either MW150 retentate (Figure 7c) or MW180 retentate (Figure 7d) showed a significant reduction in cell viability only at the concentration of 4 mg/mL and were non-cytotoxic at 2 mg/mL and below.

**Figure 8:**
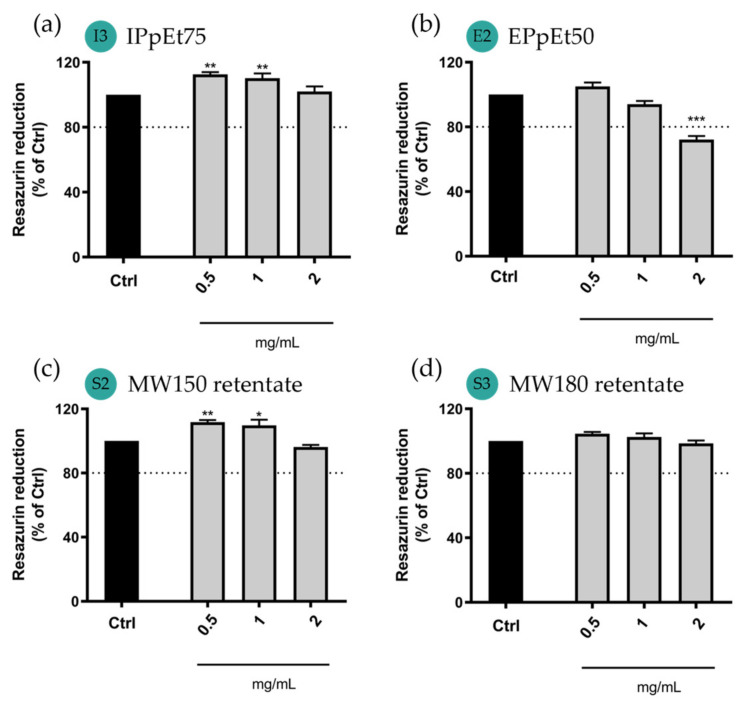
Effect of microparticle exposure for 24 h on cellular viability of macrophages (Raw 264.7 cell line) using microparticles made from (**a**) IPpEt75; (**b**) EPpEt50; (**c**) MW150 retentate; (**d**) MW180 retentate. Data correspond to the mean ± SEM of at least three independent experiments performed in duplicate and are represented as the % of untreated cells (Ctrl). Statistical analysis: one-way ANOVA with Dunnett’s multiple comparison test. Significance levels are as follows: * *p* < 0.05, ** *p* < 0.01, and *** *p* < 0.0001, compared to Ctrl.

Considering the toxicity results shown in A549 cells (Figure 7), cell viability on macrophages (Figure 8) was evaluated only at concentrations equal to or below 2 mg/mL. In Raw 264.7 cells, IPpEt75-based microparticles showed no reduction in cell viability as observed for A549 cells (Figure 8a). EPpEt50-based microparticles decreased cell viability to 72% and therefore were considered cytotoxic from this concentration upwards. The microparticles made from either MW150 (Figure 8c) or MW180 retentate (Figure 8d) showed no reduction in cell viability for all the concentrations tested, thus allowing them to be declared non-cytotoxic. 

The results obtained in both cell lines were coherent with each other and clearly showed a tendency for the microparticles prepared from galactomannan-rich fractions to promote some cytotoxicity when in high concentrations (2 mg/mL or above). This effect may be related to the difficulty in dissolving the galactomannan-rich fractions after their extraction and freeze-drying. Galactomannans may have limited solubility due to the intramolecular interactions between polymer segments, leading to aggregation and, eventually, precipitation [50]. Freeze-drying further exacerbated this effect [49] and could promote the existence of some particulate (undissolved material) when the microparticles were prepared and later when delivering the drug. The existence of particulate material has been shown to be more likely to promote inflammation and further cytotoxic effects in lung cells than the soluble polysaccharide counterpart [54]. Despite that, the results shown for the galactomannan-based microparticles are still promising, as only a higher concentration of 2 mg/mL or above showed cytotoxic effects. Both types of formulations (arabinogalactan-rich and galactomannan-rich), in concentrations of 1 mg/mL and below, were then proposed to be non-harmful for the cells present in the alveoli. 

## 4. Conclusions

This work intends to be a proof of concept that coffee polysaccharides-derived microparticles can be used as drug delivery systems, opening new avenues for the future exploitation of pulmonary insulin administration for Diabetes Mellitus treatment. In this work, both galactomannans and arabinogalactans were isolated, initially using the approach for coffee beverages’ solubilisation and for espresso and instant coffee, respectively, followed by a sequential purification methodology. From a more sustainable point of view, the polysaccharides were also extracted from spent coffee grounds (SCG), one of the main by-products of the coffee industry. The microparticles obtained by spray drying the extracts in the presence of insulin were shown to be able to carry and deliver the insulin cargo. The rate of insulin release was seen to be more dependent on the type of polysaccharide that composed the microparticle compared to its origin (espresso, instant, or SCG). Apart from EPpEt50-based ones, all microparticles were non-cytotoxic for mammalian cells at concentrations equal to or below 2 mg/mL, while EPpEt50-based microparticles were non-cytotoxic for concentrations equal to or below 1 mg/mL (*p* < 0.01). Overall, the present work demonstrates the potential of polysaccharides from coffee as drug carriers, using insulin as a model. It was also shown how a by-product could be used for such a noble purpose as the pharmaceutical application is, thus promoting a symbiotic existence between the food industry as a source of polysaccharides and pharmaceutical industries toward the development of clinical solutions that use a more sustainable use of resources. 

## Data Availability

Data is contained within the article or Supplementary Materials.

## Supplementary Materials

The following supporting information can be downloaded at: https://www.mdpi.com/article/10.3390/pharmaceutics15041213/s1, Figure S1: Insulin calibration curve; Figure S2: Absorbance profile at 405 nm of the filtered supernatants from (a) EPpEt50; and (b) IPpEt75.
