# Supplementary material for: Polysaccharide-Based Carriers for Pulmonary Insulin Delivery: The Potential of Coffee as an Unconventional Source
Source: Pharmaceutics. 2023 Apr 11;15(4):1213. doi: 10.3390/pharmaceutics15041213 (PMC10144660; doi:10.3390/pharmaceutics15041213)
Supplement: Supplementary file 1 [file pharmaceutics-15-01213-s001.zip › pharmaceutics-2277814-supplementary.pdf]

Polysaccharide-Based Carriers for Pulmonary Insulin Delivery: The Potential of Coffee as an Unconventional Source

Sara A. Valente [1], Guido R. Lopes [1], Isabel Ferreira [2,3], Miguel F. Galrinho [1], Margarida Almeida [4], Paula Ferreira [4], Maria T. Cruz [2,3], Manuel A. Coimbra [1] and Cláudia P. Passos [1,*]

[1] LAQV-REQUIMTE, Department of Chemistry, University of Aveiro, 3810-193 Aveiro, Portugal
[2] Center for Neuroscience and Cell Biology, University of Coimbra, 3004-517 Coimbra, Portugal
[3] Faculty of Pharmacy, University of Coimbra, 3000-548 Coimbra, Portugal
[4] CICECO, Department of Materials and Ceramic Engineering, University of Aveiro, 3810-193 Aveiro, Portugal
* Correspondence: cpassos@ua.pt; Tel.: +351-234-370706

Supplementary figures

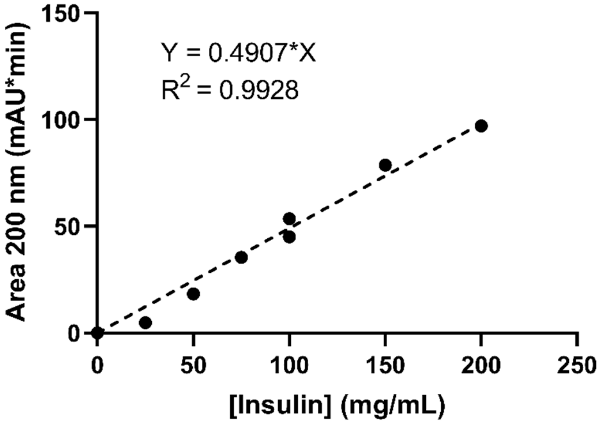

Figure S1. Insulin calibration curve.

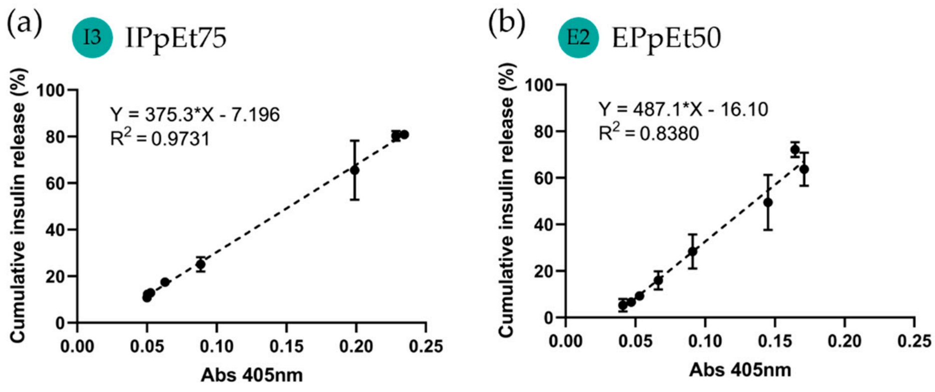

Figure S2. Absorbance profile at 405 nm of the filtered supernatants from (a) EPpEt50; and (b) IPpEt75.
